# The Survivor Unmet Needs Survey (SUNS) for haematological cancer survivors: a cross-sectional study assessing the relevance and psychometric properties

**DOI:** 10.1186/1472-6963-14-211

**Published:** 2014-05-09

**Authors:** Alix Hall, Catherine D’Este, Flora Tzelepis, Rob Sanson-Fisher, Marita Lynagh

**Affiliations:** 1 Priority Research Centre for Health Behaviour, Faculty of Health, The University of Newcastle & Hunter Medical Research Institute, Callaghan, NSW, Australia; 2 National Centre for Epidemiology and Population Health, Research School of Population Health, ANU College of Medicine, Biology & Environment, Australian National University, Acton, Canberra, ACT, Australia

**Keywords:** Haematological cancer survivors, Psychometric evaluation, Needs assessment, Psycho-oncology, Unmet needs

## Abstract

**Background:**

Relevant and psychometrically sound needs assessment tools are necessary for accurate assessment of haematological cancer survivors unmet needs. No previous study has developed nor psychometrically evaluated a comprehensive needs assessment tool for use with population-based samples of haematological cancer survivors. This study aimed to assess the validity and reliability of the Survivor Unmet Needs Survey (SUNS) with haematological cancer survivors.

**Methods:**

The relevance, content and face validity of the SUNS to haematological cancer survivors was assessed using qualitative interviews. Psychometric evaluation was conducted using data collected from haematological cancer survivors, aged 18–80 years at recruitment and recruited from four Australian cancer registries. Construct, convergent and discriminant validity; internal reliability and floor and ceiling effects were assessed. A second survey was completed by a sub-sample of survivors recruited from two of the four registries to assess test-retest reliability.

**Results:**

Results from 17 qualitative interviews confirmed the relevance, face and content validity of the original items of the SUNS for use with haematological cancer survivors. Overall, 1,957 eligible haematological cancer survivors were contacted by the cancer registries. Of these 1,280 were sent a survey, and 715 returned a survey (37% of eligible survivors contacted and 56% of survivors sent a survey). A total of 529 survivors completed all 89 items of the SUNS and were included in the exploratory factor analysis. Exploratory factor analysis supported the original five-factor structure of the SUNS. Evidence for convergent validity was established, with all five domains of the SUNS illustrating a moderate positive correlation with all three subscales of the Depression Anxiety and Stress Scale (DASS-21). All Cronbach’s alpha values were above 0.9 and all corrected item-total correlations were acceptable (>0.2). Criteria for discriminant validity was not met, with only 10 of the 15 (67%) a-priori hypotheses supported. Test-retest reliability was acceptable for 40 of the 89 items (45%) and for three of the five domains. Significant floor effects were evident for all five domains.

**Conclusions:**

The SUNS demonstrates evidence for multiple features of validity and reliability as a measure of unmet needs for haematological cancer survivors. However, evidence supporting some psychometric properties was limited.

## Background

Haematological cancers are cancers of the blood or immune system [1-3]. Research has estimated haematological cancers to be the fourth most commonly diagnosed cancer type in the economically developed world [4]. Survival rates for some haematological cancers are increasing across several countries [5-7].

Haematological cancers are a unique and diverse subgroup of cancers. They can range from chronic and incurable, to acute and aggressive [3]. Treatment is equally variable and may include “watchful-waiting”, chemotherapy, radiotherapy, immunotherapy and bone marrow transplant [3,8]. Like other cancers, haematological cancers impact on multiple aspects of a person’s life, for some resulting in tiredness and fatigue [9,10], anxiety and depression [9,11] and social impacts including limited leisure time [12], missing family and social events [13], and employment-related outcomes [13-15].

Supportive care that is responsive to cancer survivors’ physical, emotional, psychological, informational and social needs is necessary to achieve optimal cancer care [16,17]. Needs-based assessment provides a means of identifying patient concerns and the level of assistance patients perceive they need to address their concerns [18-20]. Data from needs-based assessments can be used in clinical practice to enhance patient-provider communication by efficiently identifying patient problems and directing appropriate care and services to patients [21]. Population-based assessment of needs can be used to allocate resources, improve care and direct services towards the patient-identified areas where further assistance is most needed [17,21,22].

Accurate and systematic identification of cancer survivors’ needs is necessary to ensure appropriate supportive care is provided [22,23]. Consequently, needs-based assessment tools that are comprehensive, relevant and have strong psychometric properties are essential. Previous research has used various criteria to evaluate self-report scales [24-26], including key psychometric properties such as content validity, internal consistency, criterion validity, construct validity, reproducibility, responsiveness, floor and ceiling effects and interpretability [24].

Two comprehensive, cancer-survivor specific needs assessment tools have been developed and psychometrically evaluated: (1) the Cancer Survivors’ Unmet Needs measure (CaSUN) [27], and (2) the Survivor Unmet Needs Survey (SUNS) [28]. The CaSUN was psychometrically assessed with 353 disease-free cancer survivors, diagnosed in the previous 1 to 15 years and recruited from two Australian hospitals [27]. The majority (97%) of survivors who completed the CaSUN had been diagnosed with breast, gynaecologic, colorectal or prostate cancer [27]. The CaSUN was found to have good acceptability, internal consistency and face, content and construct validity; however item test-retest was low [27]. The SUNS was psychometrically evaluated with a heterogeneous sample of 550 cancer survivors, including haematological cancer survivors, diagnosed 1–5 years previously and recruited from one Canadian cancer registry [28]. The SUNS was found to have high acceptability, item test-retest reliability, internal consistency and face, content and construct validity [28]. Neither of these tools has been psychometrically assessed for use in population-based samples of haematological cancer survivors. Given the variable nature and diverse treatment regimens of haematological cancers, the relevance and appropriateness of such tools for use with this population needs to be evaluated.

The SUNS was considered the most relevant of the two cancer survivor specific needs assessment instruments for psychometric evaluation with a population-based sample of haematological cancer survivors. Unlike the CaSUN, the SUNS illustrates acceptable item test-retest reliability and was psychometrically evaluated with a population-based sample of cancer survivors that included a larger proportion of haematological cancer survivors [28]. Despite the strong psychometric properties of the SUNS several important properties have not been assessed, including, convergent validity, discriminant validity (known-groups validity), test-retest reliability at the domain level, floor and ceiling effects, responsiveness and criterion validity.

This study aimed to evaluate the psychometric properties of the SUNS for use with adult haematological cancer survivors. This evaluation consisted of two phases: (1) qualitative evaluation of the relevance, content and face validity; and (2) quantitative survey of haematological cancer survivors for psychometric evaluation. This study also extended on the original psychometric evaluation of the SUNS by assessing convergent and discriminant validity, floor and ceiling effects and test-retest reliability at the domain level.

## Methods

### The measure

*The Survivors Unmet Needs Survey (SUNS)* measures cancer survivors’ unmet needs over the last month, using 89 items across five domains: *Financial Concerns* (11 items), *Emotional Health* (33 items), *Access and Continuity of Care* (22 items), *Information* (8 items) and *Relationships* (15 items) [28]. Each item is scored from zero (no unmet need) to four (very high unmet need) [28].

### Phase 1: Relevance, face and content validity

The relevance, face and content validity of the SUNS was assessed by two qualitative evaluations.

#### *Cultural relevance*

The wording of the SUNS was reviewed by a convenience sample of Australian researchers and members from the general population, who were known to the research team. Four items were reworded as a result of this process. The amended version of the SUNS was then reviewed by two native Canadians, who were also known to the research team. The two Canadian’s considered the changes appropriate. A summary of this process is reported elsewhere [29].

#### *Relevance to target population*

A convenience sample of 17 haematological cancer survivors (consent rate 41%) completed a semi-structured interview (average length 26 minutes) assessing their most prevalent unmet needs. Most survivors were male (59%; n = 10), diagnosed with non-Hodgkin’s lymphoma (59%; n = 10) and almost half were aged between 50 and 59 years at time of diagnosis (47%; n = 8). The interviews did not identify any additional concerns that were outside the scope of the original items; therefore no new items were included. All participants taking part in this interview study provided written informed consent. Human Research Ethics approval for this interview study was obtained from the University of Newcastle Human Research Ethics Committee and the relevant ethics committee associated with the specific cancer registry.

### Phase 2: Psychometric evaluation of the SUNS

#### Sample

A cross-sectional sample of haematological cancer survivors, aged between 18 and 80 years at time of recruitment were selected from four Australian state population-based cancer registries.

#### Procedure

The standard recruitment procedures of each registry were used. The first registry conducted a one-off cross-sectional survey. On behalf of the researchers, survivors were mailed a questionnaire package from this cancer registry [30].

The remaining three registries employed a rolling recruitment method, where survivors were identified and approached on an ongoing basis between September 2011 and July 2012. A passive clinician consent model (clinician notification) [31,32] was used by these three registries. Eligible survivors were contacted by the registries via mail unless their clinician had previously notified the registry not to contact their patient. Survivors who consented to the registry passing on their contact details to the researchers were mailed a questionnaire package from the researchers. Non-responders were mailed a second questionnaire package approximately 4 weeks later, and contacted via telephone after a further 4 weeks.

Return of a completed survey was taken as voluntary consent to participate. Ethics approval was obtained from the University of Newcastle Human Research Ethics Committee and the relevant ethics committees associated with each registry.

#### Measures

In addition to the SUNS, the following survivor information was used.

##### *Patient, disease and treatment characteristics*

For consenting survivors data relating to their age, sex, postcode or location at diagnosis, cancer type and date of diagnosis were obtained from the cancer registries. Cancer recurrence and current treatments received were collected via self-report. De-identified data for non-participants were collected from all four cancer registries and de-identified data for all participants were collected from two cancer registries and included: age-group at diagnosis, sex, postcode or location at diagnosis and cancer type. Identified data for consenting survivors from the other two registries were used for participant and non-participant comparisons. Chi-squared analyses were used to compare characteristics of participants and non-participants.

##### *Depression, anxiety and stress*

The Depression Anxiety and Stress Scale (DASS-21) is a self-report measure of depression, anxiety and stress over the past week, with three subscales each consisting of seven items [33]. Respondents rate each item on a scale from 0 (“not at all”) to 3 (“very much”) [34]. A total subscale score is calculated by summing all items in a subscale and multiplying by two [33]. A higher score represents a higher level of the emotional state. Calculation of subscale scores requires completion of at least six of the seven subscale items [35]. The DASS-21 has illustrated validity and reliability in both clinical and non-clinical populations [33,34,36,37].

### Statistical analysis

#### Construct validity

The distribution, frequencies and number of missing values for each of the 89 items of the SUNS were assessed. Items with more than 10% missing data or with more than 90% of responses occurring on only one of the response options were considered for potential exclusion.

Exploratory Factor Analysis (EFA) was used to assess the construct validity of the SUNS for use in a haematological cancer survivor population [38]. The principal factors method of EFA was employed due to the skewed distribution of the SUNS data [39,40]. The number of factors to retain was determined using the following three methods: (1) Kaiser-Guttman Criterion (Eigen value greater than one), (2) the break-point in the scree plot and (3) parallel analysis [39,40]. Multiple factor analyses were produced based on the results suggested by these three methods [40]. Results from all factor analyses were compared. The final factor structure was determined as having (i) at least 3 items per factor, (ii) factor loadings above 0.3 [39] and considered conceptually relevant [41]. The number of cross-loadings was also examined. Original psychometric evaluation of the SUNS found high correlations between several factors [28]. To allow for correlations between the factors, a promax oblique rotation was used to simplify the factor structure [39-41]. Listwise deletion removed observations with any missing data on the SUNS.

#### Convergent validity

An association between unmet needs and anxiety, depression and/or stress has previously been found in heterogeneous samples of cancer survivors [18,42-44] and, to a lesser extent, haematological cancer survivors [9]. Spearman’s rank correlations between the final factor scores of the SUNS and anxiety, depression and stress scores on the DASS-21 were calculated. Based on previous criteria used to assess the psychometric properties of self-report scales, a correlation coefficient above 0.4 was considered acceptable evidence of convergent validity [25].

#### Known-groups (discriminant) validity

Compared to their counterparts, a higher level of some unmet needs has been found for cancer survivors: reporting not being in remission (stable, recurrent and metastatic disease) [18,45]; diagnosed at a younger age [29,42,45-50]; and receiving some cancer treatments [43,45,46,51]. Based on this previous research it was hypothesized that median domain scores would be higher for: (1) survivors reporting a disease recurrence vs. survivors not reporting a disease recurrence; (2) survivors aged younger than 60 years at diagnosis vs. survivors aged 60 years or older at diagnosis; and (3) survivors currently receiving active treatment (including, chemotherapy, radiotherapy, bone marrow/stem cell transplant or harvest, hormone, antibody and targeted therapy) vs. those not receiving active treatment.

Domain scores were calculated by summing all items in a domain and dividing by the number of non-missing items for participants who answered more than 70% of items in a domain [29]. Due to the non-normal distribution of the data participant domain scores were compared by recurrence, age group at diagnosis and treatment status using the Wilcoxon rank sum test. Known-groups validity was considered acceptable if ≥ 75% of the proposed hypotheses were supported [24].

#### Internal reliability

Corrected item –total correlations above 0.2 [25,52] and Cronbach’s alpha between 0.7 and 0.95 [24] were considered acceptable indicators of internal reliability. Casewise deletion removed observations with missing data.

#### Test-retest reliability

Survivors who were recruited from two of the cancer registries, returned a completed initial survey after 4 April 2012, and consented to future contact from the researchers, were sent a second copy of the SUNS, 7–14 days after the receipt of their original survey. This time period was chosen to reduce recall bias and the likelihood of a change occurring in the participant’s level of reported unmet needs [24]. Item test-retest reliability was assessed for each item using weighted Kappa, applying quadratic weighting [53]. A kappa coefficient >0.6 was considered acceptable [54]. Test-retest reliability at the domain level was assessed using intra-class correlation coefficients (ICCs) between mean domain scores from Time 1 and Time 2. ICCs ≥0.7 were considered acceptable [24].

#### Floor and ceiling effects

The percentage of respondents reporting the lowest and highest possible scores for each domain were calculated. Domains where >15% of respondents obtained the lowest or highest possible scores were considered indicative of floor and ceiling effects [24].

### Sample size

Based on previous recommendations, a sample size of 400 survivors was considered adequate for factor analysis and estimation of reliability and validity coefficients [55-57], while a sample size of at least 50 participants has been suggested for assessing adequate levels of test-retest reliability [24].

## Results

### Response rates

A total of 1,957 eligible survivors were contacted by the cancer registries and 1,280 were sent a survey. Seven hundred and fifteen survivors returned a completed survey (37% of all eligible survivors contacted and 56% of eligible survivors sent a survey); of these, 529 (74%) completed all 89 items of the SUNS. A total of 146 eligible survivors were mailed a second copy of the SUNS; of these, 125 returned a completed survey (86%).

### Participant and non-participant characteristics

The majority of participants were diagnosed with Non-Hodgkin’s lymphoma (59%; n = 397) and most were male (n = 393; 58%). Approximately one third were aged between 60 and 69 years at diagnosis (n = 236; 35%). The median time since diagnosis was 35 months (quartile 1 = 20, quartile 3 = 51). Participants and non-participants were statistically significantly different in regards to age-group at diagnosis and cancer type (Table 1).

**Table 1:** Statistically significant differences between participant and non-participant characteristics

**Characteristics**	**Participants (n = 715)**^**a**^		**Non-participants (n = 1242)**^**a**^		**Chi-squared χ**^**2**^**(df), *p***
	**N**	**%**	**N**	**%**	
*Cancer Type*					11.05(3), *0.011*
Non-Hodgkin’s Lymphoma (NHL)	397	59%	667	56%	
Leukemia	129	19%	194	16%	
Myeloma	108	16%	203	17%	
Other lymphoma	42	6.2%	123	10%	
*Age at diagnosis*					25.12(4), *<0.001*
15-39	54	8.0%	169	14%	
40-49	71	11%	144	12%	
50-59	179	26%	263	22%	
60-69	236	35%	341	29%	
70+	136	20%	270	23%	

^a^Totals may not equal sample size due to missing values.

### Psychometric properties of the SUNS

#### Construct validity

The distributions of all items were skewed to the right. The median for all 89 items ranged from 0 (SD = 0.72) to 1 (SD = 1.28). The full range of response options were used for every item. No items had response options which included 90% or more of the distribution. Missing data for the 89 items ranged from 2.0% to 5.3%.

Five factor structures were assessed: a 7-factor model based on the Kaiser criteria; a 3, 4, 5 and 6 factor model based on the scree plot; and a 5-factor model based on the parallel analysis. After rotation, the 5-factor model was deemed most appropriate, as all factors contained more than 3 items, the highest factor loading of each item was >0.3 [39] and the structure was considered the most conceptually relevant. Cross-loadings were present for all five models.

The factor loadings of the final 5-factor model are shown in Table 2. The 5-factor model closely reflected the original factor structure of the SUNS [28]. All items from the original *Information*, *Access and Continuity of Care* and *Emotional Health* domains loaded highest on factor 5, factor 2 and factor 1, respectively. All but two of the 11 items from the original *Financial Concerns* domain loaded highest on factor 4. Two items originally from the *Financial Concerns* domain loaded more highly with all items from the *Access and Continuity of Care* domain. Twelve of the 15 items from the original *Relationships* domain loaded together on factor 3. Three items originally from the *Relationships* domain loaded highest on factor 1, with all items from the original *Emotional Health* domain. Conceptually these five items were most consistent with the original domains of the SUNS. Additionally, the factor loadings of these five items on their original domains was higher than the predetermined criterion of 0.3. Most cross-loadings in the 5-factor model occurred between items from the original *Relationships* domain cross-loading with items from the original *Emotional Health* domain. Based on statistical results and conceptual relevance, the original factor structure of the SUNS was identified as appropriate for use with haematological cancer survivors.

**Table 2:** Factor loadings for the 89 items of the Survivor Unmet Needs Survey (SUNS)

**Domains from original measure**	**Item**	**Factor 1**	**Factor 2**	**Factor 3**	**Factor 4**	**Factor 5**
**Information**	Finding information about what signs to look for and when to be concerned		0.34			**0.51**
	Knowing which sources of information to trust					**0.59**
	Finding information about all my treatment choices, including no treatment at all		0.38			**0.61**
	Finding information about complementary or alternative therapies		0.31			**0.51**
	Dealing with fears about cancer spreading	0.32				**0.62**
	Dealing with worry about whether the treatment has worked	0.33				**0.59**
	Dealing with feelings of worry (anxiety) between follow-ups	0.43				**0.55**
	Dealing with not feeling sure that the cancer has gone	0.34				**0.49**
**Financial concerns**	Worry about earning money				**0.75**	
	Having to take a pension or disability allowance				**0.76**	
	Paying household bills or other payments				**0.84**	
	Adapting to living on a pension or disability allowance				**0.82**	
	Paying non-medical costs related to my cancer (travel, accommodation, special foods, etc.)				**0.62**	
	Finding what type of financial assistance is available and how to obtain it				**0.46**	
	Finding car parking that I can afford at the hospital or clinic		0.43*		0.34	
	Understanding what is covered by my medical insurance or benefits		0.43*		0.30	
	Knowing how much time I would need away from work				**0.56**	
	Doing work around the house (cooking, cleaning, home repairs etc.)				**0.39**	
	Doing yard work (lawn mowing, etc.)	0.33			**0.39**	
**Access and continuity of care**	Finding information about who I should contact if I have a problem or concern		**0.50**			
	Finding information about cancer and its effects in a way I can understand		**0.52**			0.41
	Finding out what is involved in follow-up care		**0.54**			0.37
	Making sure my family doctor could get information from specialists		**0.65**			
	Making sure I was treated in a hospital or clinic that was as physically pleasant as possible		**0.67**			
	Having access to cancer services close to my home		**0.50**			
	Having access to cancer services at night and on weekends		**0.71**			
	Getting appointments with my family doctor quickly enough		**0.63**			
	Getting appointments with specialists quickly enough (oncologist, surgeon etc.)		**0.79**			
	Getting follow-up tests quickly enough		**0.85**			
	Getting test results quickly enough		**0.79**			
	Having access to care from other health specialists (e.g. dieticians, physiotherapists, occupational therapists)		**0.68**			
	Making sure I had choices about which hospital or clinic I could go to		**0.75**			
	Making sure health care workers had access to my medical information when planning services for me		**0.85**			
	Feeling comfortable in the waiting room		**0.63**			
	Making sure I had enough time to ask my doctor or nurse questions		**0.81**			
	Making sure all my health care workers had all the medical files related to my cancer care		**0.87**			
	Getting the health care team to attend promptly to my physical needs		**0.78**			
	Finding health care professionals who were friendly and could have a joke with me		**0.84**			
	Making sure the health care team understood and was aware of my feelings and emotional needs		**0.70**			
	Making sure I was treated like a person, not just another case		**0.77**			
	Understanding the information I was given		**0.67**			
**Relationships**	Dealing with the way other people react to my new priorities and my different outlook on life			**0.58**		
	Dealing with losses and changes in my relationships			**0.48**		
	Telling others how I was feeling physically	0.37		**0.50**		
	Telling others how I was feeling emotionally	0.42		**0.49**		
	Talking to my family and friends about how they were feeling	0.35		**0.44**		
	Finding someone to talk to who understands and has been through a similar experience	0.36		**0.45**		
	Dealing with people who expect me to be “back to normal”	0.47		**0.52**		
	Dealing with people not knowing what to say or how to behave	0.35		**0.53**		
	Dealing with people who expect me to feel happy or relieved when treatment has ended	0.49*		0.43		
	Dealing with people not understanding what I’m going through	0.61*		0.47		
	Dealing with how people are not able to cope with my illness	0.42		**0.51**		
	Dealing with people accepting that having cancer has changed me as a person	0.52		**0.54**		
	Dealing with reduced support from others when treatment has ended	0.38		**0.46**		
	Dealing with strains in relationships	0.50*		0.39		
	Finding someone to listen to me even if there is nothing they can do	0.47		**0.48**		
**Emotional Health**	Dealing with people not understanding how my physical abilities have changed	**0.54**		0.37		
	Dealing with feeling like I am a burden to my family and friends	**0.60**				
	Dealing with feeling depressed	**0.79**				
	Dealing with feeling tired	**0.73**				
	Dealing with feeling stressed	**0.76**				
	Dealing with feeling worried (anxious)	**0.78**				
	Dealing with feeling lonely	**0.61**				
	Dealing with feeling vulnerable	**0.76**				
	Dealing with worry about the emotional well-being of my family	**0.64**				
	Dealing with grief and loss	**0.70**				
	Dealing with feelings about death and dying	**0.78**				
	Dealing with not feeling able to set future goals or make long-term plans	**0.82**				
	Dealing with losing confidence in my own abilities	**0.87**				
	Dealing with feeling a loss of control	**0.91**				
	Coping with feelings of despair	**0.88**				
	Coping with feeling like a different person	**0.83**				
	Dealing with not feeling happy or relieved when treatment has ended	**0.69**				
	Dealing with not being able to feel ‘normal’	**0.84**				
	Trying to stay positive	**0.84**				
	Trying to keep a sense of hope	**0.84**				
	Dealing with feeling guilty about what I have put others through	**0.78**				
	Dealing with being told I had cancer	**0.63**				
	Wanting to reflect on what I have achieved	**0.66**				
	Dealing with not wanting to do the things I used to do	**0.83**				
	Knowing how to relax	**0.79**				
	Dealing with feelings of isolation	**0.70**				
	Coping with having a bad memory or lack of focus	**0.79**				
	Dealing with changes in how my body appears	**0.67**				
	Dealing with changes in my physical ability	**0.78**				
	Coping with going back into the ‘real’ world	**0.73**				
	Coping with things not going back to how they were before I had cancer	**0.85**				
	Dealing with missing important events like holidays	**0.70**				
	Support for finding meaning or new purpose in life	**0.73**				

*Items loaded highest on a factor that was not their original domain. However, based on conceptual relevance and that all of these items loaded >0.3 on their original domain all of these items were kept within their original domain.

#### Known-groups (discriminant) validity

As shown in Table 3, haematological cancer survivors reporting a disease recurrence compared to those not reporting a recurrence, had higher median domain scores for the *Financial Concerns*, *Access and Continuity of Care* and *Emotional Health* domains. Median scores for all five domains were higher for younger (<60 years at diagnosis) than older (≥60 years at diagnosis) survivors. Median domain scores for the *Financial Concerns* and *Emotional Health* domains were higher for survivors currently receiving treatment relative to those not receiving treatment.

**Table 3:** Median domain scores of haematological cancer survivors in relation to cancer recurrence, age at diagnosis and current treatment

	***Cancer recurrence***					***Age at diagnosis***					***Current treatment of chemotherapy, radiation, bone marrow/stem cell harvest/transplant, hormone or antibody)***				
	***Yes***		***No/unsure***			**<60 years**		**≥60 years**			**Yes**		**No**		
**Domain**	**n**	**Median**	**n**	**Median**	**p-value**^**a**^	**n**	**Median**	**n**	**Median**	**p-value**^**a**^	**n**	**Median**	**n**	**Median**	**p-value**^**a**^
*Information*	149	0.38	499	0.38	0.13	291	0.63	350	0.13	<0.001*	147	0.50	525	0.25	0.07
*Financial concerns*	151	0.36	502	0.18	0.02*	293	0.36	353	0.09	<0.001*	149	0.36	526	0.18	0.005*
*Access and continuity of care*	154	0.14	508	0.09	0.01*	295	0.14	356	0.05	<0.001*	152	0.18	532	0.09	0.05
*Relationships*	154	0.33	509	0.27	0.25	293	0.53	358	0.07	<0.001*	151	0.40	533	0.27	0.28
*Emotional Health*	155	0.68	509	0.33	0.02*	294	0.55	357	0.21	<0.001*	151	0.58	534	0.30	0.008*

*Statistically significant at 5% significance level, confirming original hypothesis for known-groups (discriminant) validity.

^a^p-value from Wilcoxon rank sum test.

#### Convergent validity

All five domains of the SUNS obtained a correlation coefficient above 0.4 with all three subscales of the DASS-21. Correlation coefficients ranged from 0.44 to 0.73.

#### Internal consistency

All corrected item-total correlations were above 0.2, ranging from 0.61 to 0.88. All five domains had high Cronbach’s alpha values (>0.9) (Table 4).

**Table 4:** Cronbach’s alpha coefficient and percentage of participants reporting the lowest and highest possible score for each of the five domains of the Survivor Unmet Needs Survey (SUNS)

**Domain**	**Number of participants answering all domain items**^**a**^	**Cronbach’s alpha**	**Number of participants answering >70% of domain items**^**b**^	**n (%) lowest score**	**n (%) highest score**	**Median (First Quartile and third Quartile)**
Information	652	0.93	686	267 (39)	0 (0.0)	0.38 (0.0, 1.14)
Financial concerns	650	0.92	689	267 (39)	2 (0.3)	0.18 (0.0, 0.82)
Access and continuity of care	647	0.97	698	285 (41)	1 (0.1)	0.09 (0.0, 0.55)
Relationships	680	0.97	698	252 (36)	3 (0.4)	0.27 (0.0, 1.07)
Emotional health	625	0.99	698	173 (25)	1 (0.1)	0.36 (0.03, 1.21)

^a^Participants answering all items in the domain and included in the calculation of Cronbach’s alpha.

^b^Participants answering >70% of items in a domain and included in the calculation of floor and ceiling effects.

#### Test-retest reliability

Weighted Kappa coefficients between item responses from Time 1 and Time 2 (mean of 28 days, SD = 16.1 days) ranged from 0.25 to 0.76 (M = 0.58; SD = 0.09). Forty items (45%) met the criteria for acceptable item test-retest reliability. ICC’s between mean domain scores at Time 1 and Time 2 ranged from 0.61 to 0.77 (*Financial Concerns* ICC = 0.61, 95% CI: 0.48, 0.71; *Access and Continuity of Care* ICC = 0.68, 95% CI: 0.57, 0.77; *Relationships* ICC = 0.72, 95% CI: 0.62, 0.79; *Information* ICC = 0.73, 95% CI: 0.63, 0.81; *Emotional Health* ICC = 0.77, 95% CI: 0.68, 0.83).

#### Floor and ceiling effects

Floor effects were observed for all five domains. Few ceiling effects were observed (Table 4).

## Discussion

The study results provide initial support for the SUNS as a suitable measure of unmet needs in adult haematological cancer survivors. Qualitative interviews with members of the target population verified the relevance of the items and supported the face and content validity of the measure. Quantitative analyses demonstrated strong psychometric properties, across multiple aspects of validity and reliability. However, evidence for some areas of reliability and validity were limited and require further investigation.

The original 5-factor structure of the SUNS was deemed most statistically and conceptually appropriate for use with haematological cancer survivors. However, the results from the factor analysis showed several discrepancies relative to the structure of the original SUNS. In particular three items originally from the *Relationships* domain loaded highest with items from the original *Emotional Health* domain. The majority of cross-loadings were also found between items from the original *Relationships* domain with items from the *Emotional Health* domain. It is likely that emotional and relationship needs are highly related; these domains had a high correlation (r_s_ = 0.848) in the original psychometric evaluation of the SUNS [28]. Future research should further evaluate the appropriateness of the 5-factor structure of the SUNS for use with haematological cancer survivors.

While most (10 out of 15; 67%) of our hypotheses relating to known-groups validity were supported, the scale did not meet the specified criteria for acceptable known-groups validity [24]. Three of our five hypotheses relating to the expected differences in domain scores between survivors receiving treatment, compared with those not receiving treatment, were not supported. It is possible that only specific treatment types affect cancer survivor unmet needs. For instance, previous studies have found an association between cancer survivor unmet needs and chemotherapy [43,45,46] and radiotherapy [45,46,51] treatment, while a study of multiple myeloma survivors found no difference in the supportive care needs of survivors who had received a bone marrow transplant compared to those who did not receive this treatment [9]. We were unable to compare the unmet needs of survivors by individual treatment types due to the small number of survivors currently receiving each individual treatment type.

Internal consistency was high with all corrected item-total correlations above 0.6 and all Cronbach’s alpha values above 0.9. However, it is possible that the high Cronbach’s alpha values reflect some redundancy within the scale’s items [24]. This seems particularly likely for the domains of *Access and Continuity of Care*, *Relationships* and *Emotional Health,* which all demonstrated Cronbach’s alpha values above the recommended criteria of 0.95 [24]. This finding is consistent with the results from the original psychometric evaluation of the SUNS [28]. The Cronbach’s alpha values for these three domains may have also been inflated due to the large number of items (≥15) within each of the domains [24]. Consequently, a shorter scale may be warranted. At 89 items, this seems reasonable, particularly if this scale is to be routinely applied in research and clinical practice. Development of the Short Form SUNS (SF-SUNS) with a large, heterogeneous sample of Canadian cancer survivors has recently been conducted [58]. It is recommended that future studies assess the validity and reliability of the SF-SUNS with haematological cancer survivors.

Unfortunately, the majority of items (55%) illustrated low test-retest reliability. These items may lack stability and therefore may not be appropriate for detecting changes in these needs over time. However, the mean Kappa statistic for items in this study was higher than the mean Kappa statistic reported for item test-retest of the CaSUN (0.58 vs. 0.13) [27], which has previously been used to assess the unmet needs of haematological cancer survivors [9,49]. Additionally, test-retest reliability at the domain level was not acceptable for two domains. It is possible that the time frame between survivors returning the initial survey and test-retest survey (mean = 28 days) was too long. As the SUNS assesses unmet needs over the past month, it is possible that survivors experienced a true change in the level of some of their unmet needs. Further research utilising a shorter time period between initial and follow-up surveys is needed to confirm the test-retest reliability of the SUNS for haematological cancer survivors.

Floor effects were present for all five domains, indicating that most survivors were experiencing low levels of unmet needs across all five domains. Consequently, the ability of the SUNS to detect improvements in haematological cancer survivors’ unmet needs may be impaired, thus impacting on the responsiveness of the scale [24]. Floor effects are not uncommon for cancer specific needs assessment measures [18,52]. However, this may not be a limitation of the scale but may instead be an accurate reflection that many cancer survivors are in fact doing well and have few unmet needs. This is a possibility, with almost a quarter (21%) of Australian and Canadian haematological cancer survivors reporting no unmet needs on all 89 items of the SUNS in a previous study conducted by the authors [29]. Alternatively, the floor effects may also be influenced by possible sampling bias, as survivors experiencing a high level of unmet needs may be less likely to participate in research.

We conducted a thorough and rigorous psychometric evaluation of the SUNS. However, there were some limitations. Due to the cross-sectional design, the predictive validity and responsiveness of the scale were unable to be assessed. Future research should conduct longitudinal studies with haematological cancer survivors to confirm these other psychometric properties of the SUNS. Criterion validity was also not assessed due to the lack of a gold standard needs assessment tool. Despite the inclusion of a qualitative component to our psychometric evaluation, survivors were not asked to review the relevance of each individual item of the SUNS. Instead, an open-ended interview was conducted to discuss survivors’ most prevalent/important unmet needs. To further strengthen the content validity of the SUNS for this population, the relevance of each individual item should be confirmed via direct feedback from haematological cancer survivors.

Study limitations include the low response rate (37% of all eligible survivors contacted and 56% of all eligible survivors sent a survey). However this is consistent with previous registry-based studies that have reported low response rates of 49% of a large heterogeneous sample of Canadian cancer survivors [28], 43% of a sample of non-Hodgkin’s lymphoma survivors [59], 41% of a large heterogeneous sample of Australian cancer survivors [45], 34% of a large heterogeneous sample of America cancer survivors [60] and 8% of adolescent and young adult cancer patients [31]. In an effort to overcome this limitation, multiple efforts were made to increase the response rates of haematological cancer survivors approached for this study. Strategies included assessing the effectiveness of a tailored invitation letter to improve response rates to survivors recruited from Registry A [30], and the inclusion of several evidence-based strategies for increasing questionnaire response rates for survivors recruited from Registries B, C and D, such as the use of multiple reminders to non-responders and reducing the length of the survey sent to survivors [61]. In addition, non-participant and participant age at diagnosis and cancer type were statistically significantly different. These limitations may affect the generalizability of these results to the entire population of haematological cancer survivors.

## Conclusions

Needs-based assessment is a vital step in achieving optimal supportive care. However, no previous multi-dimensional needs assessment tool has been psychometrically evaluated for use with a population-based sample of haematological cancer survivors. The present study fills this gap by identifying the SUNS as a suitable measure of unmet needs in haematological cancer survivors. This study also extends on the original psychometric evaluation of the SUNS by assessing additional psychometric properties, including convergent and known-groups validity, and floor and ceiling effects. In the future, large-scale longitudinal studies should assess responsiveness, predictive validity and test-retest reliability, and also confirm the factor structure of the SUNS with haematological cancer survivors.

## Abbreviations

SUNS: Survivor Unmet Needs Survey; DASS-21: Depression Anxiety and Stress Scale 21 items; CaSUN: Cancer Survivors’ Unmet Needs measure; EFA: Exploratory factor analysis; SD: Standard deviation; ICC: Intra-correlation coefficient; SF-SUNS: Short Form Survivor Unmet Needs Survey.

## Competing interests

The authors declare that they have no competing interests.

## Authors’ contributions

AH and RSF developed the aims for this manuscript. AH, FT and ML assisted in organizing data collection. AH and FT developed, conducted and oversaw data cleaning procedures. AH and CD conducted statistical analysis. All authors contributed to the interpretation of the results. AH wrote and prepared the manuscript. All authors reviewed, edited and approved the manuscript.

## Authors’ information

AH is a PhD student investigating the unmet needs of haematological cancer survivors. She has previous experience in the area of cancer survivorship. CD is a professor in biostatistics. She has over 18 years of experience in health research and teaching. FT is a post-doctoral research fellow. She was awarded a 3-year Leukaemia Foundation of Australia/Cure Cancer Australia Foundation Fellowship (2011–2013), to investigate haematological cancer survivors’ perceptions of quality of care. RSF is a Laureate Professor of Health Behaviour with over 20 years of experience and an order of Australia award. RSF has research expertise in a number of different areas in health behaviour, including: addressing the psychosocial needs of cancer patients and improving health outcomes for vulnerable populations. ML is a senior lecturer and researcher in the area of health behaviour. ML’s research interests include: health behaviour change, tobacco control and assessment of unmet needs of haematological cancer survivors and their support persons.

## Pre-publication history

The pre-publication history for this paper can be accessed here:

http://www.biomedcentral.com/1472-6963/14/211/prepub
